# Microemulsion Formulation of Carbendazim and Its *In Vitro* Antifungal Activities Evaluation

**DOI:** 10.1371/journal.pone.0109580

**Published:** 2014-10-13

**Authors:** Pengfei Leng, Zhiming Zhang, Qian Li, Maojun Zhao, Guangtang Pan

**Affiliations:** 1 Maize Research Institute, Sichuan Agricultural University, Ya’an, PR China; 2 Chongqing College of Humanities, Science and Technology, School of Foreign Languages, Hechuan District, Chongqing, PR China; 3 College of Life and Basic Sciences, Sichuan Agricultural University, Sichuan Ya’an, PR China; Korea University, Republic of Korea

## Abstract

The fungus *Rhizoctonia solani* Kuhn is a widespread and destructive plant pathogen with a very broad host range. Although various pathogens, including *R. solani*, have been traditionally controlled using chemical pesticides, their use faces drawbacks such as environmental pollution, development of pesticide resistance, and other negative effects. Carbendazim is a well-known antifungal agent capable of controlling a broad range of plant diseases, but its use is hampered by its poor aqueous solubility. In this study, we describe an environmentally friendly pharmaceutical microemulsion system using carbendazim as the active ingredient, chloroform and acetic acid as solvents, and the surfactants HSH and 0204 as emulsifiers. This system increased the solubility of carbendazim to 30 g/L. The optimal microemulsion formulation was determined based on a pseudo-ternary phase diagram; its physicochemical characteristics were also tested. The cloud point was greater than 90°C and it was resistant to freezing down to −18°C, both of which are improvements over the temperature range in which pure carbendazim can be used. This microemulsion meets the standard for pesticide microemulsions and demonstrated better activity against *R. solani* AG1-IA, relative to an aqueous solution of pure carbendazim (0.2 g/L). The mechanism of activity was reflected in the inhibition of against *R. solani* AG1-IA including mycelium growth, and sclerotia formation and germination were significantly better than that of 0.2 g/L carbendazim water solution according to the results of *t*-test done by SPSS 19.

## Introduction

In recent years, there has been an increase in the prevalence of fungal infections of crops worldwide. *R. solani* is a particularly widespread, soil-borne plant-pathogenic fungus which causes seed and hypocotyl rot, collar rot, root rot, damping off and wire stem in many plant species [1]–[4]. One of the most well-known symptoms of *R. solani* infection is black scurf on potato tubers, corresponding to the sclerotia of the fungus. The anastomosis group of *R. solani*, AG1-IA, is a major pathogen in Latin America, causing Sheath Blight (SB) on rice and Banded Leaf Sheath Blight (BLSB) on maize, particularly in Venezuela [5]. Soybean yield losses of up to 35% have been attributed to Rhizoctonia foliar blight (RFB), with potential yield reductions of up to 70% [6]. Chemical pesticides have contributed significantly towards controlling crop diseases, including those caused by *Rhizoctonia*. An evaluation of the inhibitory effects of 18 types of fungicides towards *R. solani* AG1-IA in vitro and in vivo revealed that mycelial growth was strongly inhibited and disease symptoms could be alleviated to a large extent [7]. However, pesticide resistance, environmental pollution and other negative effects are attributed to the overuse of pesticides. Worldwide, it is estimated that about 2.5 million tons of pesticides are used on crops annually, with the environmental costs associated with pesticide overuse approximating $100 billion annually [8]. The risk of fungicide resistance increases when a single chemical pesticide is used intensively for an extended period [9]. Consequently, the biological control of *R. solani* with antagonists has been examined in detail [10]–[12], although such methods are strongly influenced by environmental conditions. For example, a preparation of *Trichoderma harzianum* significantly reduced the incidence of bean diseases caused by both *Sclerotium rolfsii* and *R. solani*, but its biocontrol capacity was inversely correlated with temperature [13]. This supports the requirement to develop more efficient methods of pesticide delivery, thereby minimizing the application rates of the active pesticide ingredient. Microemulsions of pesticides thus represent a relatively environmentally friendly formulation that decrease the use of organic solvents and increase the wettability, dispersibility, and penetration ability of the pesticide droplets. A microemulsion is defined as “a colloidal dispersion composed of oil phase, aqueous phase, surfactant and co-surfactant at appropriate ratios, which is a single optically isotropic and thermodynamically stable liquid solution” [14], [15]. Our previous study on a difenoconazole/propiconazole microemulsion indicated that the usage of xylene was reduced by 75%, while the use of toluene in a triadimefon microemulsion was reduced by 70%, relative to commercial formulations [16], [17]. Studies have indicated that the control efficiency of pesticide microemulsions is superior to those of ordinary emulsions, wettable powders, and other pesticide formulations [16], [17]. It has been reported that a microemulsion of λ-cyhalothrin possesses better performance and stability than its commercial formulation [18]. Similarly, a microemulsion of triadimefon displayed significantly better antifungal activity against *R. solani* AG1-IA, when compared to a 20% triadimefon emulsion [17].

Carbendazim is a well-known antifungal agent that can be used to control a broad range of diseases on field crops, fruits, and vegetables, including sclerotinia rot of canola, wheat head blight, peanut leaf spot, and SB on rice [19]. Carbendazim is fairly easily degraded through chemical, physical and biological processes, such as UV, H_2_O_2_ and microorganisms [20]–[22]. It has been officially registered in several countries and is used extensively in controlling diseases in field and greenhouse crops, and in forests. A mixture of flusilazole and carbendazim has been recommended for soybean rust control [23]. Another study revealed that carbendazim was the most effective fungicide for inhibiting the mycelial growth of *Moniliophthora. pernicious* [24]. Unfortunately, its solubility in water is 6.11 µg/mL and it possesses a high melting point, making it difficult to apply [25].

The aim of the present study was to establish a pharmaceutical microemulsion system for the application of carbendazim, using chloroform and acetic acid as co-solvents, the surfactants HSH and ‘0204’ as emulsifiers, and methanol as a co-surfactant. The optimal microemulsion formulation was selected based on testing the dilution stability, low and high temperature stabilities, freezing stability, and other physiochemical parameters. Finally, the antifungal activities of the optimal carbendazim microemulsion formulation against *R. solani* AG-IA were assessed.

## Materials and Methods

### Materials

#### Chemicals

Carbendazim was purchased from Shandong Zouping Agrochemical Co. Ltd (Zouping, Shandong, China). A pure carbendazim standard was purchased from the Chinese CRM/RM Information Center. The emulsifier HSH and the commercially used agricultural emulsifier 0204 (Hai’an Petrochemicals, Jiangsu, China) were of commercial grade. Dyes Sudan IV and methylene blue were of analytical grade and supplied by Sinopharm Group Chemical Reagent Co. Ltd. (Shanghai, China). Methanol, chloroform, and acetic acid were purchased from Sichuan Xilong Chemical Co. Ltd (Chengdu, Sichuan, China). Deionized water was used in all experiments.

#### Microorganism

*Rhizoctonia solani* AG1-IA and *Alternaria alternata* (Fr.) Keisskr were maintained on potato dextrose agar medium (PDA: potato, 200****g; dextrose, 20****g; agar, 20****g; deionized water, 1****L) at 28**°**C.

### Solvent and surfactant screening

#### Solvent screening

Pure carbendazim was placed in different test tubes, and its solubility at various temperatures was assessed in 10****mL different solvents. The solvent that completely dissolved carbendazim was selected for subsequent experiments; the solution was maintained at 0°C to prevent the formation of a precipitate or phase inversion. The solution got from this is the “Oil” will be used the next steps.

#### Surfactant assay

In order to determine the most effective surfactant(s), microemulsion formulations were devised by maintaining the contents of various surfactants equal to that of the active ingredient (carbendazim). The microemulsion was then initially placed in a water bath at 20°C for 24****h. The quality of each surfactant was then determined according to the appearance of the samples, the temperature range over which transparency was maintained, and low temperature and freezing stability.

### Pseudo-ternary phase diagram construction

Pseudo-ternary phase diagrams were constructed using the SAA (optimal formulation of emulsifier HSH, agricultural emulsifier 0204 and methanol got from the solvent and surfactant screening part) titration method; first, a carbendazim compound solution was prepared using a mixture of the emulsifier HSH, agricultural emulsifier 0204 and methanol (4: 2: 1, w/w/w) [26]. Nine ratios of the carbendazim compound solution and SAA (1: 9, 2: 8…9: 1, w/w) were dispersed in vials. To each vial, water was added drop-wise until a critical change in transparency was observed. The amount of water required to induce this change was measured for each of the nine vials; this experiment was conducted in triplicate. The mean value was used for constructing a pseudo-ternary phase diagram using the software Origin 7.5. Based on the proportions of carbendazim, emulsifiers, water and other components, an optimal formulation was finally determined.

### Physicochemical characteristics

#### Microemulsion type

The appearance, centrifugation properties, staining reactions, and dilution characteristics, as described in our previous work, were used to characterize the obtained microemulsion [16].

#### HPLC analysis

A Shimadzu (Kyoto, Japan) liquid chromatograph, equipped with an LC-10 AT VP solvent pump unit and an SPD-10A VP UV-Visible detector, was used to analyze the optimal carbendazim microemulsion indicated in the previous section. Samples were injected manually through a 10 µL loop with a Rheodyne injector. Separations were conducted at room temperature on a C18 VP-ODS, 250×4.6****mm column with a mobile phase of methanol/0.1% ammonium hydroxide (65/35, v/v), with a flow rate of 1.0****mL/min. Carbendazim was detected at 280****nm [27]. A standard solution of carbendazim was injected to construct a stable calibration curve. The microemulsion was injected in triplicate, after which the concentration of carbendazim was calculated according to the following formula:
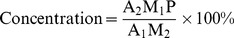
Where: A_1_: peak area of standard carbendazim; A_2_: peak area of the tested microemulsion sample; M_1_: the weight of standard carbendazim (g); M_2_: the weight of the tested microemulsion sample (g); P: purity of standard carbendazim (%).

#### Physiochemical stability

The following four aspects were evaluated: emulsion stability, low temperature stability, reaction to freezing, and turbidity point. Three types of microemulsion formulations were assayed, differing in the type of water used: distilled water, water containing known concentrations of Ca^2+^ and Mg^2+^ (182****mg/L each), or a standard hard water solution (342****mg/L total dissolved solids) [16], [28].

### Antifungal activity assay

#### Toxicity bioassay

Assay of the inhibition against *R*. *solani* AG1-IA was conducted based on a previous study, with some modifications [29]. A mixture of carbendazim microemulsion and PDA medium was prepared, with a 0.2****g/L solution of carbendazim in water as a positive control and PDA medium with no carbendazim as a negative control. Microemulsions with carbendazim concentrations of 0.05, 0.1, 0.2, 0.4 and 0.8****mL/L and aqueous solutions with carbendazim concentrations of 1, 2, 4, 8 and 16****mL/L were assayed in this experiment. All of the test plates were inoculated with healthy *R*. *solani* AG1-IA isolates with a colony diameter of 0.4****cm. The plates were incubated in the dark at 28°C and the diameters of the fungal colonies were measured every 12****h. Subsequently, the inhibition ratios were calculated, while regression analysis was used to determine the EC_50_ and EC_90_ values. Each treat was performed in three biological replicates. EC_50_ and EC_90_ were got according to regression equation. The inhibitory effects of the carbendazim microemulsion and solutions were calculated as indicated below.
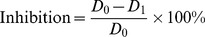
Where: D_0_: colony diameter in the negative control; D_1_: colony diameter in the treatment.

Another antifungal activity assay through mycelial growth inhibition with *A. alternata* was conducted as indicated above. Microemulsions with carbendazim concentrations of 0.4, 0.8, 1.6, 3.2 and 6.4 mL/L and aqueous solutions with carbendazim concentrations of 8, 16, 32, 64 and 128 mL/L were analyzed. The plates were incubated in the dark at 25°C and the diameters of the fungal colonies were measured 7 days later.

#### Sclerotia formation

Plates with carbendazim microemulsion, at concentrations of 0.05, 0.1, 0.2, 0.4 and 0.8****mL/L, were respectively compared with those with the positive control, at concentrations of 1, 2, 4, 8 and 16****mL/L (recorded as contrast groups A, B, C, D and E, respectively), following inoculation with *R*. *solani* AG1-IA. After incubation for 15 days at 28°C, sclerotia were collected and dried to a constant weight at 80°C. The inhibition of sclerotia formation by the carbendazim microemulsions and 0.2****g/L carbendazim aqueous solutions were calculated with respect to the negative control. Each experiment was conducted in triplicate.

#### Sclerotia germination

The mature sclerotia of *R. solani* AG1-IA were dipped in carbendazim microemulsions (0.05, 0.1, 0.2, 0.4 and 0.8****mL/L) or in aqueous carbendazim solutions of 0.2****g/L (1, 2, 4, 8 and 16****mL/L) (the respective comparison groups were recorded as A, B, C, D and E, respectively) for 30****seconds and then air-dried. Sclerotia were dipped in sterile water as a negative control. Treated sclerotia were inoculated onto untreated PDA plates and incubated at 28°C for 24****h. Each treatment consisted of 30 sclerotia and was repeated in triplicate.

### Statistical analysis

All the regression functions were performed by Microsoft Excel (Redmond, WA, USA) and the other statistical analysis was conducted using SPSS 19.0 software (SPSS, Chicago, IL, USA); a *t*-test was used to analyze the inhibitory effects of the carbendazim microemulsion and solutions.

## Results and Discussion

### Components of carbendazim microemulsion

Carbendazim solubility was limited in all of the solitary solvents screened. However, our analysis suggested that a combination of acetic acid and chloroform could be used as an effective solvent. When chloroform and acetic acid were mixed in a volumetric ratio of 3: 2, the solubility of carbendazim attained 30 g/L, which was significantly (*P*<0.001) higher than its highest known solubility in solitary solvents. Buffers, co-solvents, surfactants, and complexants are the most commonly used excipients to improve the solubility of a nonpolar drug or pesticide [30]. Our results suggest the value of experimentally testing the solubility of such compounds in mixed solvents. For subsequent experiments, the concentration of carbendazim was maintained at 20 g/L.

As shown in Table 1, the emulsifier HSH and the agricultural emulsifier 0204 were best-suited for use in carbendazim microemulsion. Therefore, they were mixed in gravimetric ratios of 1: 4, 2: 4, 3: 4, 4: 4, 4: 3, 4: 2 and 4: 1 to determine the best formulation. The optimal ratio (by weight) of the emulsifier HSH to the agricultural emulsifier 0204 was 4: 2; this sample exhibited the best low temperature stability and thermal stability (Table 2). This phenomenon may be explained by the formation of novel polymers of these compounds. Many studies have indicated that a blend of two emulsifiers is more efficient than a single substance, given comparable hydrophilic–lipophilic balance (HLB) values [31], [32]. In particular, one study indicated that the maximum stability of oil-in-water emulsions using a mixture of the commercial surfactants Tween and Span was achieved at a certain molar ratio of these two surfactants [31]. Others have indicated that block copolymers prefer to localize to the interface, which significantly reduces the interfacial tension [33], [34]. Research on oil-in-water emulsion systems stabilized with non-ionic surfactant blends revealed the presence of thin aqueous films between the oil phases and oil droplets, coalescing against their homophase [35].

**Table 1:** Surfactant screening assay.

Surfactants	Appearance	Appearance after 24 h
**Agricultural emulsifier 500**	White emulsion	Evident destabilization
**Agricultural emulsifier 700**	White emulsion	Evident destabilization
**Agricultural emulsifier 0201**	White, turbid	Destabilization, crystallization
**Agricultural emulsifier 0204**	Semitransparent solution	Slight destabilization
**Agricultural emulsifier 0207**	White, turbid	Destabilization, crystallization
**Agricultural emulsifier 1600**	White, turbid	Destabilization, crystallization
**Agricultural emulsifier 2201**	Semitransparent solution	Destabilization, crystallization
**By-140**	White, turbid	Destabilization, crystallization
**Emulsifier HSH**	Transparent solution	Transparent
**Emulsifier OP-10**	White turbid	Destabilization, crystallization
**Tween40**	White turbid	Destabilization, crystallization

**Table 2:** Results of the surfactant screening assay.

Emulsifier HSH: agricultural emulsifier 0204	Appearance	Appearance after 24 h	Low temperature stability	Cloud point (°C)
**1∶4**	Turbid	–	–	–
**2∶4**	Turbid	–	–	–
**3∶4**	Transparent	Transparent	Some precipitation	>54
**4∶4**	Transparent	Transparent	Transparent	>54
**4∶3**	Transparent	Transparent	Transparent	>54
**4∶2**	Transparent	Transparent	Transparent	>54
**4∶1**	Turbid	–	–	–

“–”: not tested.

### Pseudo-ternary phase diagrams and preparation of microemulsion

**Figure 1:**
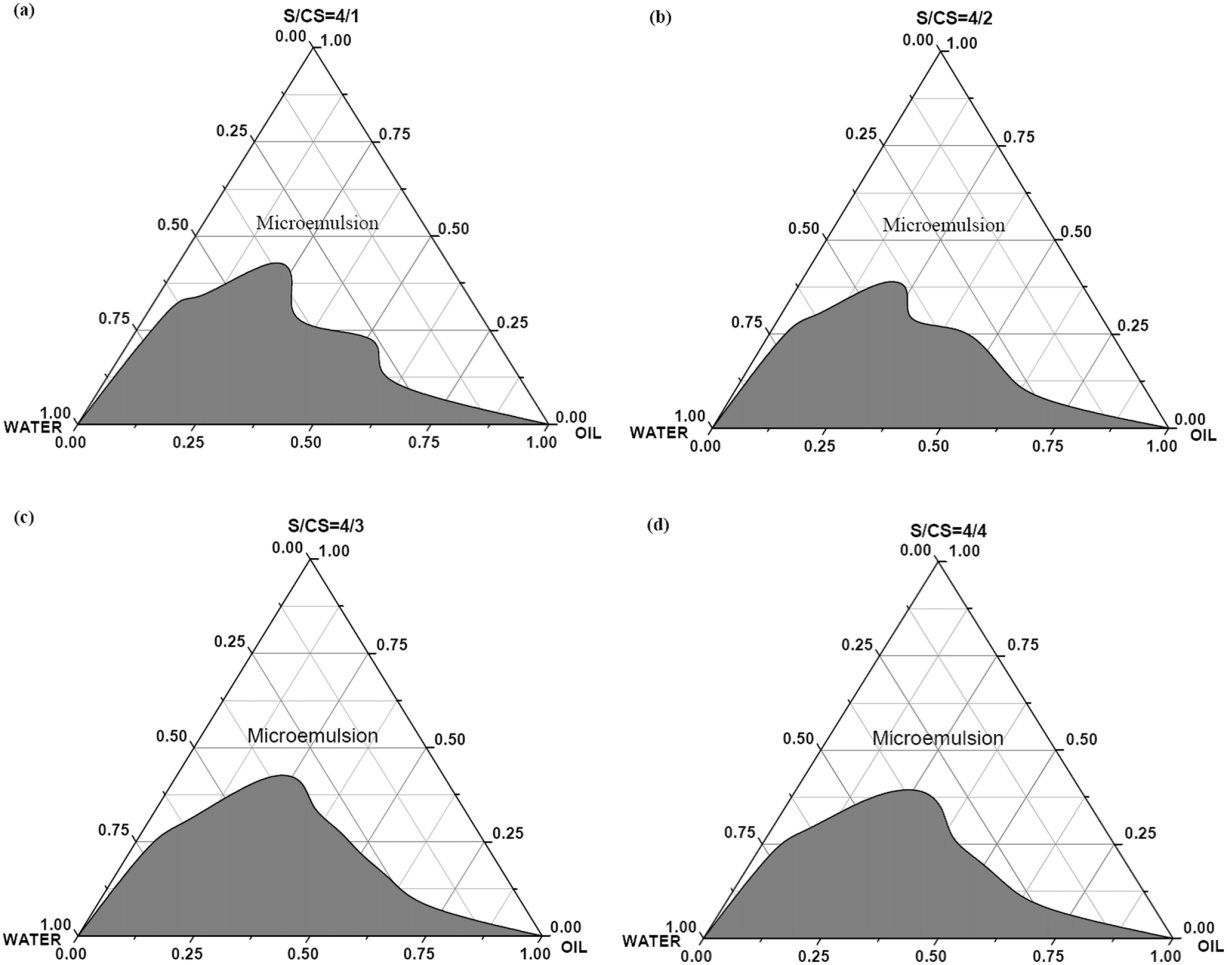
Pseudoternary phase diagrams of carbendazim microemulsion using different ratios of emulsifiers/methanol: (a) 4: 1, (b) 4: 2, (c) 4: 3, (d) 4: 4.

**Figure 2:**
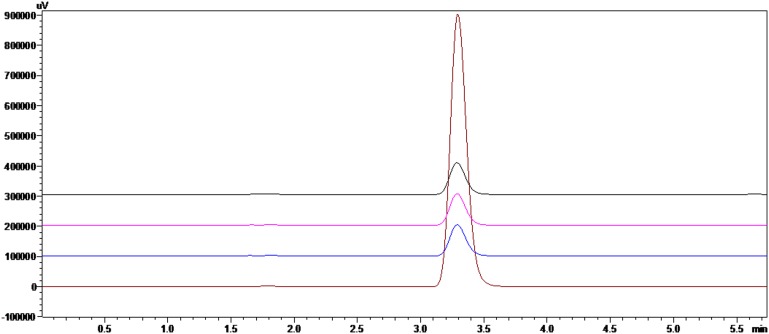
Chromatograms of a carbendazim standard and carbendazim microemulsion samples. HPLC conditions are described in the text. Note: Chromatography of the carbendazim standard (top) and microemulsion samples (bottom) were repeated in triplicate.

Pseudo-ternary phase diagrams are presented in Figure 1a–d. A smaller microemulsion region (39.26%) was formed when the surfactants/methanol ratio was 4: 1 (Figure 1a). As shown in Figure 2b, when this ratio was decreased to 4: 2, the proportion of the mono-phase region decreased (36.57%). When the ratio was reduced further to 4: 3 (Figure 1c), a larger mono-phase region was observed, accounting for 40.39% of the area in the phase diagram. Finally, decreasing the ratio to 4: 4 (Figure 1d) diminished the proportional area of this microemulsion to 37.01%. Therefore, a surfactant: methanol ratio of 4: 3 was selected for preparing subsequent microemulsion. Candidate microemulsion with certain proportions of Oil, SAA and water were designed according to the pseudo-ternary phase diagram indicated in Figure 1c. Their characteristics were assessed (Table 3) and the optimum formulation was determined to be as follows: Oil/SAA/water = 1: 3: 4; SAA consisted of HSH, the agricultural emulsifier 0204, and methanol, with a surfactant: methanol ratio of 4: 3.

**Table 3:** Assay of carbendazim microemulsion formulations.

Oil/SAA/H_2_O	Appearance	Freezingexperiment	Lowtemperaturestability	Heatstoragestability	Cloud point (°C)
**1/4/5**	Transparent,Tyndall effect	Froze, recoveredupon thawing	Transparent, noprecipitate	Transparent	72
**1/3/4**	Bluish transparent,Tyndall effect	Did not freeze, lowviscosity	Transparent, noprecipitate	Transparent	>90
**2/5/3**	Bluish transparent,Tyndall effect	Did not freeze butbecame turbid	Precipitate	Transparent	>90
**2/3/3**	Transparent,Tyndall effect	Turbidity andcrystallization observed	Precipitate	Transparent	>90

Next, we assessed the formation of the microemulsion at room temperature. As indicated in Figure 1, the proportional area of the microemulsion in the corresponding phase diagrams first decreased and then increased as the ratio of methanol increased. These results are comparable with those of a previous study, the authors of which suggested that the increase in the isotropic phase area indicates an increased incorporation of solvent (in their case, ethanol) into the surfactant film, thereby leading to a decrease in the bending modulus of this film [36]. The addition of short-chain alcohols to non-ionic oil/water microemulsion decreases the bending energy [37]. Methanol, as a co-surfactant, plays an important role in microemulsion formulations, as it reduces the interfacial tension and bending energy, adjusts the HLB value, and is easily inserted into the structure of the surfactant. As the relative proportion of methanol increased, the liquidity and appearance of the microemulsion improved noticeably. Our previous research on triadimefon microemulsion revealed that the addition of methanol, up to a certain limit, significantly increased the area occupied by the microemulsion in the corresponding phase diagram and also improved its appearance [17].

### Physiochemical detection

#### Type of carbendazim microemulsion

The carbendazim microemulsion exhibited bluish transparent fluorescence and did not form any precipitate, which is typical of most microemulsions. In view of the colloidal properties of microemulsions, we tested the appearance and occurrence of the Tyndall effect in this microemulsion. Directing monochromatic light through it clearly produced a beam of light. Furthermore, no phase separation was observed after centrifugation at 8000**** *g* for 20****min at room temperature. These results are consistent with the results of Zhong [38] and our previous studies [16], [17]. These results may be additionally explained by the theory of mutual dissolution.

#### Carbendazim concentration in the microemulsion

As shown in Figure 2, the retention time of carbendazim was 3.29****min, with good peak symmetry at 280****nm and high peak purity. The mean concentration of carbendazim was 1.19%, with a standard deviation and coefficient of variation of 0.006 and 0.60%, respectively. The analysis method was precise, suggesting that it has potential in future assays of carbendazim content in microemulsion or other matrices.

#### Physiochemical stability assays

The physiochemical stability of the microemulsion was assessed in triplicate as follows.

*Dilution stability*: Carbendazim microemulsion was diluted with standard hard water to concentrations of 5%, 2%, 1% and 0.5%. All samples remained transparent and uniform; no floating oil or precipitate was observed.

*Low temperature stability*: After storage at 0°C for 14 days, there was no precipitate formation or phase separation; no significant change in the mobility or stability was observed.

*Freezing experiment*: The sample did not freeze or destabilize after storage for 7 days at −18°C. This is a particularly noteworthy characteristic of the microemulsion, since it permits the application of carbendazim at very low temperatures if necessary.

*Cloud point*: This was determined as the temperature at which the sample became observably turbid; this experiment was conducted by increasing the temperature of the water bath at 2.0°C/min with gentle stirring of the immersed sample. The turbidity point occurred at temperatures greater than 90°C.

The useful characteristics of the carbendazim microemulsion assessed in this study may be explained by the unique interaction between the co-solvents, resulting in enhanced solute stability as a function of the balance between thermodynamics and kinetics. A previous study reported that a λ-cyhalothrin microemulsion formulation exhibited acceptable transparency over a broad temperature range, and superior performance and stability compared to other commercial formulation [18].

When using ionic surfactants, phase transition occurs as the concentrations of electrolytes change. We prepared microemulsion using distilled water, naturally hard water, and a standard sample of hard water, using the same formula; physiochemical testing revealed that there were no differences among these methods. This is particularly surprising with respect to the turbidity point (>90°C), a parameter that is easily affected by water hardness. This finding is different from what has been reported by Song [39] and in previous studies [16], [17]. This may potentially be explained by the turbidity point of carbendazim being higher than 90°C, with the differences between the three types of water being too small to produce observable effects. Although there were no obvious changes in the physiochemical stabilities of the microemulsions, it is possible that slight reduction in these factors occurred when hard water was used. The concentrations of Ca^2+^ and Mg^2+^ often affect the HLB values of the surfactants in microemulsion systems [39], [40]. These changes may include thinning of the interface film, an increase in the sizes of oil droplets a decrease in the uniformity of their distribution, and an increased rate of particle collisions, thereby leading to phase separation at high temperatures.

### Antifungal activities

#### Inhibition of *R. solani* mycelial growth

18.13% of mycelial growth was inhibited after the *R. solani* were cultivated for 36****h in the presence of 0.05****mL/L of carbendazim microemulsion, which was significantly higher than that of a 1****mL/L aqueous solution of carbendazim (6.23%) (*P*<0.001). When the concentration of the carbendazim microemulsion was increased to 0.2****mL/L, the inhibition ratio went to 44.63%, significantly higher than that of a 4****mL/L carbendazim aqueous solution (17.32%). About 91% inhibition ratio was tested when *R. solani* were treated with 0.8****mL/L microemulsion and was significantly higher than that of 16****mL/L aqueous solution of carbendazim (60%) (*P*<0.001). With these inhibition ratios (Table 4), regression functions were then formed. The EC_50_ and EC_90_ values of the carbendazim microemulsion after 36****h of treatment were 0.20****mL/L and 0.91****mL/L, while those of a 0.2****g/L aqueous solution of carbendazim were 14.06****mL/L and 100.91****mL/L, respectively.

**Table 4:** Inhibition of *R. solani* AG1-IA mycelial growth at 36 h after treatment with carbendazim.

Treatment	Regressionfunction	Relation coefficient	EC_50_/(mL/L)	EC_90_/(mL/L)
**Carbendazim** **microemulsion**	y = 1.9266x+6.3607	0.9585	0.20a	0.91a
**0.2 g/L Aqueous** **carbendazim solution**	y = 1.4972x+3.2813	0.952	14.06b	100.91b

#### Inhibition of *A. alternata* mycelial growth

Cultivation for 7 days with the presence of 0.4 mL/L of carbendazim microemulsion resulted in a mycelial inhibition ratio of 37.1%. This was significantly higher than that of a 8 mL/L aqueous solution of carbendazim (3.7%) (*P*<0.001). When the concentration of the carbendazim microemulsion was quadrupled to 1.6 mL/L, the inhibition ratio was 65.6%, which was significantly higher than that of a 32 mL/L aqueous solution of carbendazim (23.1%). Treatment with the microemulsion at a concentration of 6.4 mL/L revealed an inhibition ratio was 96.5%, which was significantly higher than that of a 16 mL/L aqueous solution of carbendazim (60.4%) (*P*<0.001). Regression equations were then obtained using these inhibition ratios (Table 5). The EC_50_ and EC_90_ values of the carbendazim microemulsion after 7 days of treatment were about 0.8 mL/L and 4.4 mL/L, respectively, while those of a 0.2 g/L aqueous solution of carbendazim were almost 80 mL/L and 392 mL/L, respectively.

**Table 5:** Inhibition of *A. alternata* mycelial growth at 7 days after treatment with carbendazim.

Treatment	Regression function	Relation coefficient	EC_50_/(mL/L)	EC_90_/(mL/L)
**Carbendazim microemulsion**	y = 1.7582x+5.1597	0.9433	0.81a	4.35a
**0.2 g/L Aqueous carbendazim solution**	y = 1.8527x+1.4769	0.9728	79.73b	392.01b

The aforementioned results confirmed that the carbendazim microemulsion had a significantly stronger inhibitory effect on mycelial growth than did an aqueous solution of carbendazim. A reason for this observation may be that carbendazim in the form of a microemulsion could easily permeate the fungal tissues and inhibit the polymerization of free tubulin molecules by binding an arginin residue of the β-tubulin subunit and acts by disrupting cell division through linking to the nuclear spindle, which inhibits fungal growth. A microemulsion system allows the pesticide to easily overcome capillary resistance due to a decrease in the interfacial tension. Additionally, many traditional chemical and pharmaceutical products are more effective when mixtures of solvents are employed, as opposed to a single solvent [41].

#### Inhibition of *R. solani* sclerotia formation

**Figure 3:**
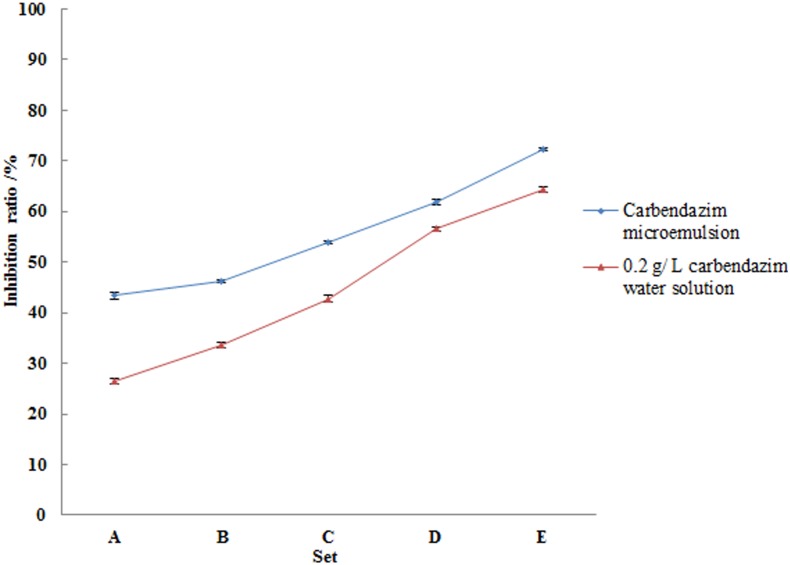
Comparisons of the inhibitory effects of the tested carbendazim microemulsions and the positive control (0.2 g/L aqueous solution of carbendazim): (a) 0.05 mL/L microemulsion and 1 mL/L water solution, (b) 0.1 mL/L microemulsion and 2 mL/L water solution, (c) 0.2 mL/L microemulsion and 4 mL/L water solution, (d) 0.4 mL/L microemulsion and 8 mL/L water solution, (e) 0.8 mL/L microemulsion and 16 mL/L water solution.

After cultivation for 15 days, sclerotia of *R. solani* were collected, and dried to constant weight; subsequently, the inhibition ratios of different carbendazim formulations were assessed (Figure 3). When the concentration of the carbendazim microemulsion was 0.05****mL/L, the inhibition ratio was 43.33%, significantly higher than the 26.43% obtained using a 1****mL/L aqueous solution of carbendazim (*P*<0.001). Sclerotial formation was further inhibited (53.83%) when the concentration of the microemulsion was increased to 0.2****mL/L, significantly higher than that obtained using a 4****mL/L aqueous solution of carbendazim (*P*<0.001). When the concentration of the microemulsion was increased to 0.8****mL/L, the inhibition ratio was 72.30%, relative to 64.30% obtained using a 16****mL/L aqueous solution of carbendazim (*P*<0.001). This may be attributed to the fact that the improved solubility of carbendazim results in a steeper gradient of the pesticide between the treatment liquid and the fungal tissues, thereby increasing the velocity of its transport into the organism’s cells. Regression equations were obtained based on the inhibition ratios (Table 4). The EC_50_ and EC_90_ values of the carbendazim microemulsion after 36****h were 0.12****mL/L and 12.03****mL/L, respectively, while those of a 0.2****g/L aqueous solution of carbendazim were 5.84****mL/L and 181.50****mL/L, respectively.

#### Inhibition of *R. solani* sclerotial germination

**Figure 4:**
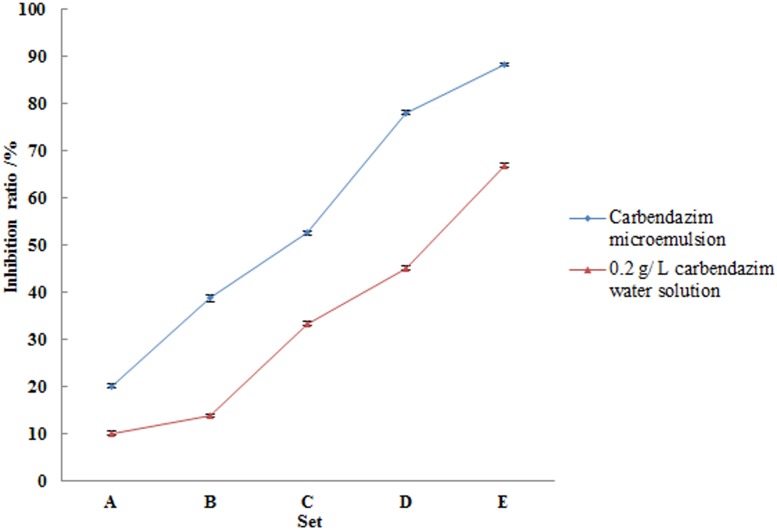
*R. solani* AG1-IA sclerotia germination rate at 24 h after treatment with carbendazim microemulsions and aqueous solutions.

Inhibition of sclerotial germination is vital in preventing crop diseases; mature sclerotia are the main sources of diseases attributed to *Rhizoctonia*. The inhibition ratios of the microemulsion were calculated, as shown in Figure 4. The rate of sclerotial germination decreased as the concentration of carbendazim increased. When the sclerotia were incubated for 24****h, increases in the strengths of both the microemulsion and aqueous solutions of carbendazim resulted in an inhibition of sclerotial germination. As observed for the inhibition of mycelial growth and sclerotia formation, carbendazim in the form of a microemulsion was consistently more effective than that in the form of aqueous solutions. At the lowest application rates, the inhibition ratio of the microemulsion was 20.03%, compared to 10.03% obtained using an aqueous solution of carbendazim (*P*<0.001). The discrepancies between the two types of treatments increased as the concentration of carbendazim increased. The inhibition ratios was 88.23% when the concentration of the microemulsion was 0.8****mL/L, compared to 66.9% obtained using a 16****mL/L aqueous solution of carbendazim. The EC_50_ and EC_90_ values of the carbendazim microemulsion after 36 hours were 0.16****mL/L and 8.76****mL/L, respectively, while those of a 0.2****g/L aqueous solution of carbendazim were 0.89****mL/L and 65.73****mL/L, respectively.

The aforementioned results are similar to those reported by Wang, who suggested that tebuconazole is more effective at inhibiting sclerotial germination than mycelial growth [42]. Based on the results of the antifungal activity assays, the application of carbendazim in the form of microemulsions should be aimed at controlling diseases, as opposed to preventing them.

In this study, we successfully developed an effective and conveniently used carbendazim microemulsion system, with improved inhibitory activity against mycelial growth and sclerotia formation in *R. solani* AG1-IA. Notably, this microemulsion was resistant to storage at −18°C with no solidification or disruption of stability occurring; similarly, it was physically stable at temperatures up to and including 90°C. This improved formulation may prove to be invaluable in controlling pathogens sensitive to carbendazim. However, further studies should be conducted to evaluate the efficacy of carbendazim microemulsion in controlling BLSB on maize, SB on rice, and other Rhizoctonia diseases at the field scale.
